# Confined Silver Nanoparticles in Ionic Liquid Films

**DOI:** 10.3390/molecules28073029

**Published:** 2023-03-28

**Authors:** Alexandre C. P. M. Alves, Luís M. N. B. F. Santos, Margarida Bastos, José C. S. Costa

**Affiliations:** CIQUP, Institute of Molecular Sciences (IMS), Department of Chemistry and Biochemistry, Faculty of Science, University of Porto, Rua do Campo Alegre s/n, P4169-007 Porto, Portugal

**Keywords:** ionic liquids, vapor deposition, thin films, microdroplets, silver nanoparticles

## Abstract

This work reports the formation of silver nanoparticles (AgNPs) by sputter deposition in thin films of three different ionic liquids (ILs) with the same anion (bis(trifluoromethylsulfonyl)imide) and cation (imidazolium), but with different alkyl chain lengths and symmetries in the cationic moiety ([C_4_C_1_im][NTf_2_], [C_2_C_2_im][NTf_2_], and [C_5_C_5_im][NTf_2_]). Ionic liquid (IL) films in the form of microdroplets with different thicknesses (200 to 800 monolayers) were obtained through vacuum thermal evaporation onto glass substrates coated with indium tin oxide (ITO). The sputtering process of the Ag onto the ILs when conducted simultaneously with argon plasma promoted the coalescence of the ILs’ droplets and the formation, incorporation, and stabilization of the metallic nanoparticles in the coalesced IL films. The formation/stabilization of the AgNPs in the IL films was confirmed using high-resolution scanning electron microscopy (SEM) and UV-Vis spectroscopy. It was found that the IL films with larger thicknesses (600 and 800 monolayers) were better media for the formation of AgNPs. Among the ILs used, [C_5_C_5_im][NTf_2_] was found to be particularly promising for the stabilization of AgNPs. The use of larger IL droplets as capture media was found to promote a better stabilization of the AgNPs, thereby reducing their tendency to aggregate.

## 1. Introduction

The synthesis and stabilization of metal nanoparticles (MNPs) resulting in a controlled size, size distribution, and shape in liquid samples is a difficult task, although crucial in order to use their unique properties. The formation of MNPs using ionic liquid (IL) media has been usually achieved by chemical reactions in ionic liquids (ILs); however, other methods have recently been considered and used as an alternative [1,2,3,4,5,6]. The sputter deposition technique, which belongs to the group of physical vapor deposition (PVD) processes, is one of the most-used methods for metal deposition [7,8]. Sputtering is particularly relevant for the formation of MNPs in different liquids due to the simplicity and speed of the process, together with the observation that uniform nanoparticles (NPs) are obtained [9,10,11]. In contrast with chemical and electrochemical techniques [12,13,14,15,16] that often necessitate additional purification stages and the addition of stabilizing agents, the sputter-deposition method enables the production of NPs in a single step, resulting in the formation of extremely pure nanoparticles. MNPs produced through sputter deposition in liquids may display unique characteristics that have potential applications in diverse fields, such as electronics, sensors, biomedicine, and catalysis, among others [17,18,19,20]. In this technique, the capture medium used must have low vapor pressure, thus making it ideally suited for ILs, since almost all ILs have negligible vapor pressure even at high temperatures [20,21,22,23,24,25,26,27,28]. ILs have several properties attractive for various applications: they usually have melting points below 100 °C, are transparent in the visible light region, and show low solvation, high viscosity, high thermal stability, ionic conductivity, and unusual miscibility behavior [29,30,31,32,33,34,35,36]. For the formation of MNPs by sputtering, molecules, atoms, or small clusters are ejected from the metal target and deposited on the substrate, which in this study was an IL sample. The ILs serve not only as media to capture the metal atoms but also as stabilizers during the formation of the NPs, as they tend to form aggregates of small and uniform sizes. When ILs are used as a stabilizing medium for the synthesis of MNPs, the properties may differ depending on the anion, cation, and size of the cation’s alkyl side chain in the IL [1,2,3,4,5,6,37,38,39,40,41,42,43,44,45,46,47]. The synthesis of MNPs in ILs is usually carried out with ILs in the bulk state, regardless of the deposition method used for the NPs. Recently, it was shown that ILs can find many applications, not only in the bulk state but also in the form of films deposited on certain substrates, such as transparent conductive oxides, metal surfaces, and carbon nanostructures, among others [21,22,23,24,48,49,50,51,52,53,54,55]. Ionic liquid films have a wide range of important applications in various fields, and their unique properties make them attractive for use in many different technologies, including as lubricants, electrolytes in batteries and supercapacitors, as effective catalysts in a range of chemical reactions, and as sensors and biosensors [20,21,22,23,24,25,26,27,46,49,54]. Few studies concerning the formation of MNPs in IL films can be found in the literature. Only Dupont et al. demonstrated the fabrication of surface-clean gold nanoparticles (AuNPs) confined in IL films [56]. To the best of our knowledge, the study of the deposition of silver nanoparticles (AgNPs) in IL films obtained using vapor-deposition approaches as we describe and show in the present paper has never been carried out. Costa et al. previously described the formation of thin films of ILs obtained by the vacuum thermal evaporation of different imidazolium-based ILs and showed that these films are composed of droplets that can differ in size, shape, and contact angle, depending on the structure of the cation–anion pairs, as well as on the roughness and chemical nature of the substrate [21,22,23,24]. Due to their negligible volatility, it is possible to characterize IL coatings/films using techniques based on reduced pressure conditions, such as scanning electron microscopy (SEM). This technique was also essential in this work to study the morphological and topographical properties of the IL films before and after the sputtering process using Ag. We used a high-resolution and reproducible physical-vapor-deposition approach to prepare very stable and homogeneous IL films, composed of droplets. This approach made it possible to control the first step in the process of formation of AgNPs in these films. The coalescence of the droplets was promoted by a plasma (argon) surface treatment of the IL’s surface during the silver deposition [22].

**Figure 1:**
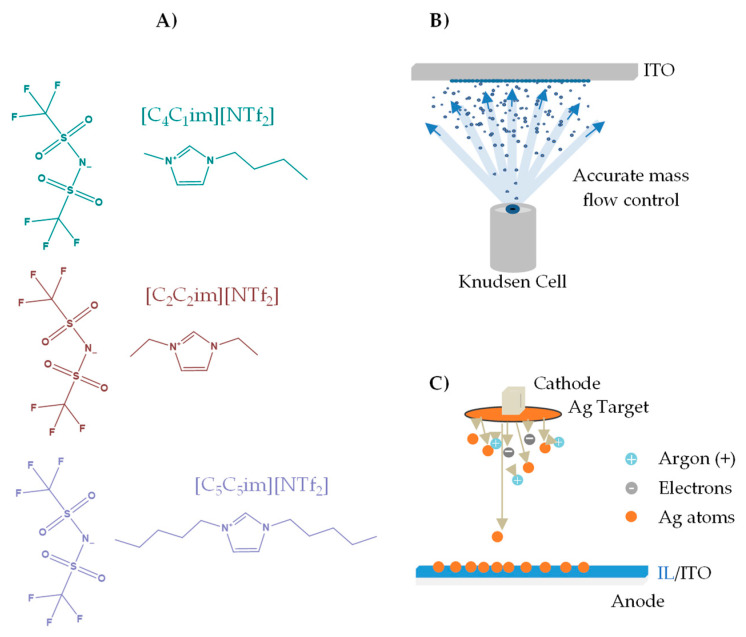
Molecular structure of the studied ionic liquids (**A**): 1-butyl-3-methylimidazolium bis(trifluoromethanesulfonyl)-imide [C_4_C_1_im][NTf_2_]; 1,3-diethyl-imidazolium bis(trifluoromethanesulfonyl)-imide [C_2_C_2_im][NTf_2_]; 1,3-dipenthyl-imidazolium bis(trifluoromethanesulfonyl)-imide [C_5_C_5_im][NTf_2_]. Schematic representation of the PVD/Knudsen effusion process of ionic liquids (**B**). Schematic representation (out of scale) of the sputter deposition of Ag onto ionic liquid films (**C**).

ILs based on imidazolium cations are one of the most studied IL families and their effectiveness in stabilizing MNPs has been previously demonstrated [3,39,43,57,58]. Association with relatively weakly coordinating anions such as tetrafluoromethanesulfonate (NTf_2_) can lead to physicochemical properties suitable for the formation and stabilization of MNPs [57,58]. The IL cations consist of a polar zone, where most of the electrostatic charge is concentrated, and a nonpolar alkyl side chain, which can vary in size and affect the properties of the ILs. For ILs with relatively large alkyl side chains (especially for imidazolium-based ILs), these chains can segregate and form nonpolar domains, while the other parts of the IL form a polar domain [30]. The formation of these domains affects the dissolution of various substances in ILs, as well as the stabilization of MNPs [59,60]. It has been reported for ILs in bulk state that the variation of these alkyl side chains can affect the MNPs’ size [47,61,62]. Based on this knowledge, we decided to investigate the thin films of three different imidazolium-based ILs with different alkyl-side-chain sizes (Figure 1) as possible media for the formation and stabilization of AgNPs. These ILs were selected to understand the impact of the alkyl-side-chain length on the stabilization and growth of AgNPs. SEM was used to obtain high-resolution images of the micro- and nanodroplets of the ILs, as well as of the coalesced IL films incorporating silver particles after the sputtering method. Finally, UV-Vis spectroscopy was used to verify and confirm the presence of AgNPs in the IL films, inferring from their characteristic surface plasmon resonance (SPR) peak. The presence of an intense band in the region of 400–420 nm indicated the presence of AgNPs in the IL films/coatings [46,63]. The UV-Vis characterization can thus give information about the existence of these AgNPs in the inner regions of the film, where their presence is not easily detected by SEM.

In brief, the major focuses of this study were to demonstrate the possibility of fabricating ILs thin films decorated with AgNPs, of stabilizing them by changing the type and morphology of the underlayer IL, and of characterizing them in detail using SEM and UV-Vis.

## 2. Results

**Figure 2:**
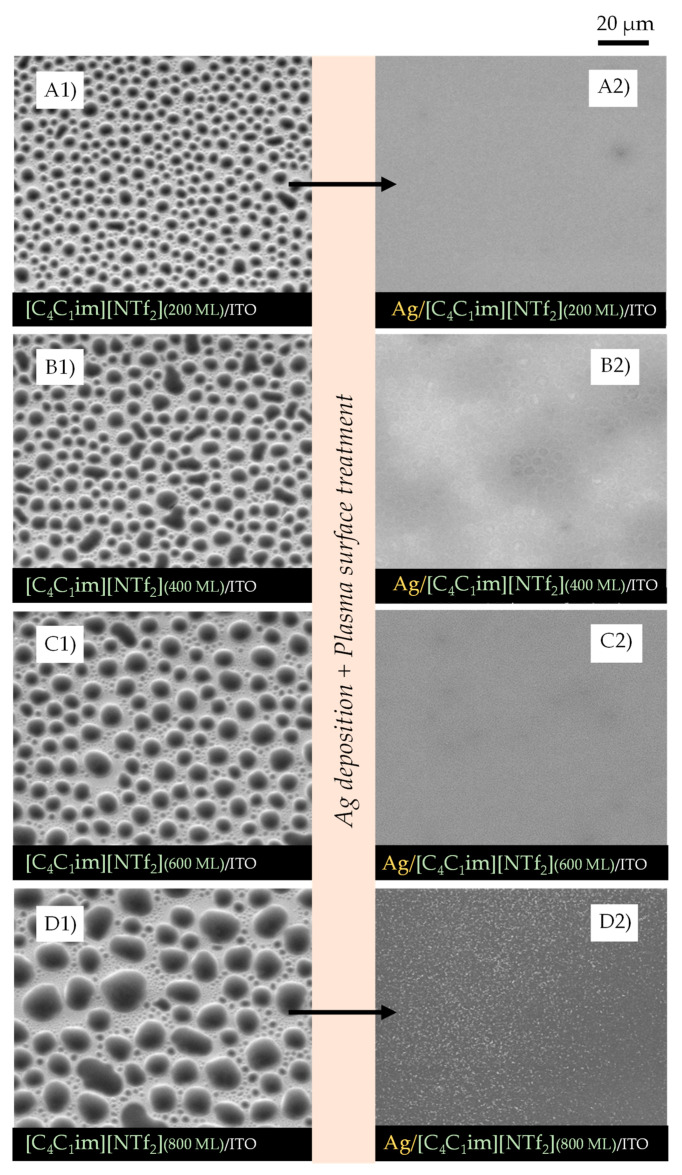
Morphology of micro- and nanodroplets for [C_4_C_1_im][NTf_2_] films with different thicknesses (200, 400, 600, 800 ML) deposited by PVD on ITO/glass surfaces (**A1**–**D1**). Morphology of the same samples after exposure to argon plasma and the deposition of AgNPs through sputtering for 40 s using a discharge current of 20 mA (**A2**–**D2**). Micrographs were acquired through high-resolution SEM using an SE detector. Top views obtained with a magnification of 2000×.

**Figure 3:**
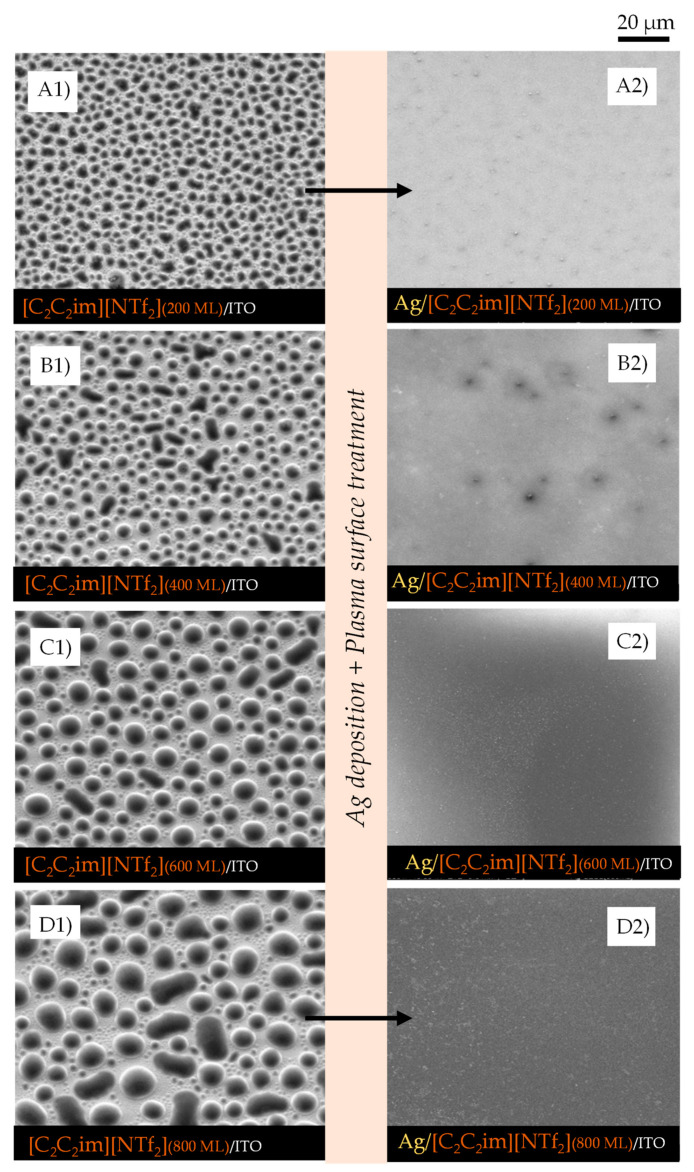
Morphology of micro- and nanodroplets for [C_2_C_2_im][NTf_2_] films with different thicknesses (200, 400, 600, 800 ML) deposited by PVD on ITO/glass surfaces (**A1**–**D1**). Morphology of the same samples after exposure to argon plasma and the deposition of AgNPs through sputtering for 40 s using a discharge current of 20 mA (**A2**–**D2**). Micrographs were acquired through high-resolution SEM by using an SE detector. Top views obtained with a magnification of 2000×.

**Figure 4:**
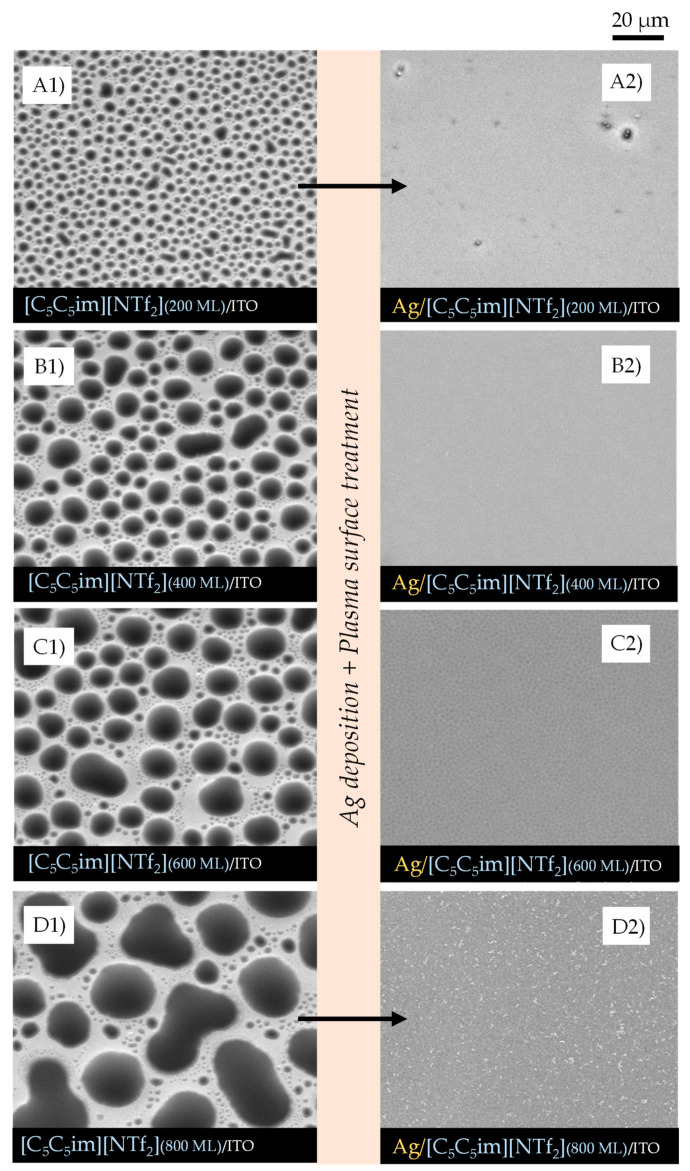
Morphology of micro- and nanodroplets for [C_5_C_5_im][NTf_2_] films with different thicknesses (200, 400, 600, 800 ML) deposited by PVD on ITO/glass surfaces (**A1**–**D1**). Morphology of the same samples after exposure to argon plasma and the deposition of AgNPs through sputtering for 40 s using a discharge current of 20 mA (**A2**–**D2**). Micrographs were acquired through high-resolution SEM by using an SE detector. Top views obtained with a magnification of 2000×.

IL films with different thicknesses (200, 400, 600, and 800 monolayers, ML) were obtained by vacuum thermal evaporation onto indium tin oxide (ITO)/glass surfaces, that were thereafter exposed to argon plasma and Ag bombardment to investigate the potential of IL films as good substrates and stabilizers for the formation of AgNPs. The successive increase in the IL film’s thickness allowed us to investigate the main differences in IL-droplet formation in terms of size, shape, and size distribution. By maintaining the same amount of Ag deposited by sputtering in each IL film surface, and by varying ILs and thicknesses, we were able to assess which IL films were more compatible and capable of being a medium for stabilizing AgNPs. Figure 2, Figure 3 and Figure 4 show the high-resolution SEM micrographs (the top views were obtained using a secondary electron detector, SE) for 200, 400, 600, and 800 ML of each IL surface before (left images) and after (right images) the plasma surface treatment and Ag deposition.

The vapor deposition of ILs onto the ITO surface leads to the formation of thin films consisting of micro- and nanodroplets (left images). These droplets are formed through mechanisms of nucleation and coalescence as described in detail in previous studies from our group [21,22,23,24]. Two steps of coalescence (1st- and 2nd-order mechanisms) lead to the formation of droplets of different sizes in the same film. A schematic representation of the typical processes of nucleation and growth of ILs is presented in Figure S4. A detailed SEM image of the ITO/glass surface is presented in Figure S5.

The first order of coalescence is a process between two or more native droplets, while the second order of coalescence occurs between already coalesced droplets [23]. The affinity of each IL with the underlayer substrate determines how well the droplets will be distributed and whether they have a non-uniform or a uniform shape (where they are rounder). Increasing the thickness of the IL film leads to an increase in the size of the micro- and nanodroplets, as has already been reported [21,22]. Figure 2A1–A4, Figure 3A1–A4, and Figure 4A1–A4 highlight this effect for [C_4_C_1_im][NTf_2_], [C_2_C_2_im][NTf_2_], and [C_5_C_5_im][NTf_2_], respectively. The minimum free area of nucleation (MFAN) and the coalescence processes are dependent on the film’s thickness [21]. In previous works, we have consistently shown the formation of ionic liquid droplets on ITO surfaces, which strongly indicates a 3D growth of the film rather than the formation of a continuous film [21,22,23,24]. For imidazolium-based ILs, the formation of microdroplets, with contact angles between 15 and 20 degrees, has been proven [21]. Only post-deposition treatments can induce a total coalescence and consequent formation of two-dimensional IL films [21,48,64].

**Figure 5:**
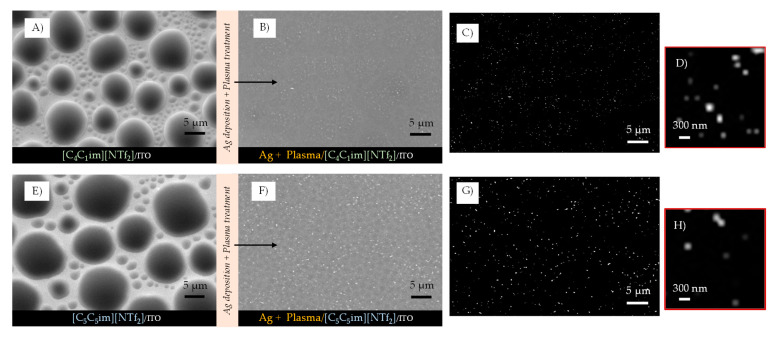
Detailed morphology of as-deposited micro- and nanodroplets (**A**,**E**) and their morphology after surface treatment with argon plasma and deposition of Ag (images (**B**,**F**)). The images correspond to the surface of [C_4_C_1_im][NTf_2_] (**A**,**B**) and [C_5_C_5_im][NTf_2_] (**E**,**F**) with a thickness of 600 ML. Images (**C**,**D**,**G**,**H**) show the presence of AgNPs in the IL films. These images were obtained by processing SEM micrographs using ImageJ software. The micrographs were acquired through high-resolution SEM using both SE (**A**,**E**) and BSE detectors (**B**,**F**).

**Figure 6:**
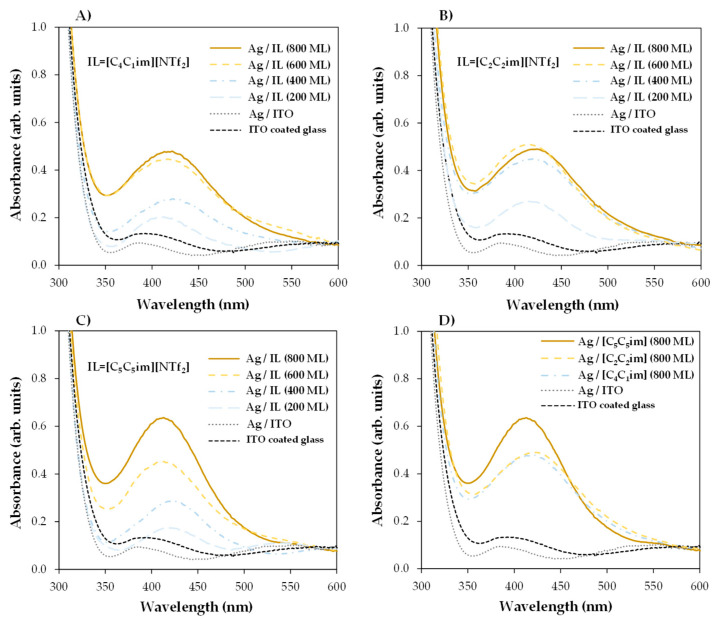
UV-Vis absorption spectra of IL films treated with argon plasma and Ag particles. Spectra for 200, 400, 600, and 800 ML of [C_4_C_1_im][NTf_2_] (**A**), [C_2_C_2_im][NTf_2_] (**B**), and [C_5_C_5_im][NTf_2_] (**C**). Comparison of the graphs obtained for each IL for a thickness of 800 ML (**D**). Spectra for ITO/glass and Ag/ITO/glass are presented for comparison. AgNPs incorporated into the IL films through 40 s of sputter deposition from a silver magnetron target and argon plasma (inducing the droplet coalescence) using a discharge current of 20 mA (≈13 nm of Ag); micro- and nanodroplets ILs previously prepared by vacuum thermal evaporation onto ITO-coated glass substrates. A single absorption peak with 400–420 nm might be assigned to the SPR band that is attributed to the presence of AgNPs in their metallic form.

By changing the thickness of the IL, we were able to gather valuable information about the processes that occur during the deposition of AgNPs by sputtering. The coalescence of the droplets due to the surface treatment with argon plasma occurred at the very beginning of the sputter-deposition process. The different shapes and size distributions of the IL droplets had a strong influence on the dispersion and/or aggregation of the AgNPs during the deposition. The progress in the deposition of Ag directly onto the surface of the ITO/glass substrate led to the formation of a metallic film since a complete aggregation of Ag particles could be observed (Figure S6). The IL films can be used as confining media to avoid Ag aggregation. According to our data, at lower thicknesses (200 ML), the aggregation of NPs was still promoted, and very thin Ag films seem to have been easily formed on the IL film’s surface (Figure 2A2, Figure 3A2 and Figure 4A2). Larger thicknesses are more favorable for the formation/stabilization of AgNPs. Reflected polarized light microscopy (PLM) images showing the surface of a Ag film (formed on the top of an IL film with low thickness), as well as the surface of a thicker IL film incorporating AgNPs, are presented in Figure S7. The PLM images of the thicker IL films exhibit the characteristic yellow color of the AgNPs. These results are explained by the larger size of the droplets that under surface treatment with argon plasma coalesce more slowly and thus encapsulate the AgNPs during deposition. This resulted in better dispersion rather than an agglomeration of the AgNPs, as seen in images (C2) and (D2) of Figure 2, Figure 3 and Figure 4. The SEM images of the ILs at 800 ML show this phenomenon even more clearly. With larger droplets, the IL is more able to behave as a confining agent, stabilizing the MNPs. Contrary to those observed for 200 ML, the Ag particles are not so prone to aggregating on the surface of the IL film. The SEM images presented in Figure 2, Figure 3, Figure 4 and Figure 5 were obtained using the electron secondary detector, which revealed detailed information on the topography of the sample’s surface. Figure 5 presents detailed SEM images of the surface of the [C_4_C_1_im][NTf_2_] and [C_5_C_5_im][NTf_2_] films incorporating Ag particles. Additional SEM micrographs obtained using both a secondary electron detector (SE) and a backscattered electron detector (BSE) are presented in Figure S8. Upon comparing both images in Figure S8, it can be strongly inferred that the white spots correspond to the AgNPs. The size distribution of the AgNPs is presented in Figure S9. The results were obtained by processing SEM micrographs using the ImageJ software. The modal diameter of these NPs was found to be lower than 60 nm. For larger thicknesses (600 and 800 ML) of IL, most AgNPs were encapsulated in the inner regions of the IL film and could not be detected by the SEM methodology. A more accurate evaluation of the formation/stabilization of AgNPs in IL films was made using UV-vis spectroscopy (Figure 6). The UV-Vis characterization confirmed the formation of AgNPs, and the results are consistent with those reported in the literature for AgNPs synthesized by other methods [46,65,66,67]. A single absorption peak between 400 and 420 nm is characteristic of the SPR band, which indicates the presence of AgNPs in their metallic form [46,65].

**Figure 7:**
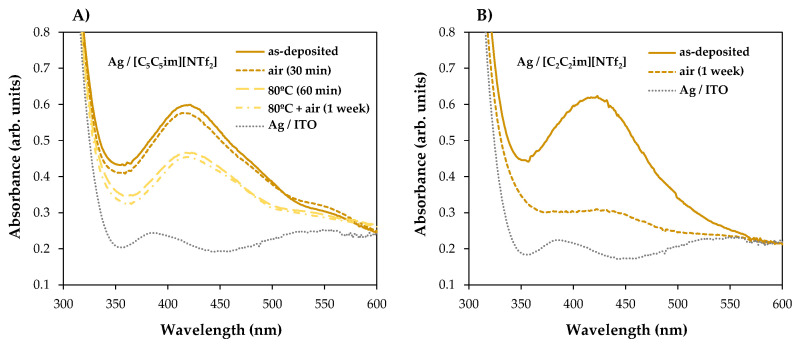
UV-Vis absorption spectra of IL films treated with argon plasma and Ag particles. Spectra for AgNPs/[C_5_C_5_im][NTf_2_] (**A**) and AgNPs/[C_2_C_2_im][NTf_2_] (**B**) exposed at different conditions: as-deposited sample; sample exposed to air for 30 min; sample annealed at 80 °C; sample annealed at 80 °C and exposed to air for 1 week. Spectra for Ag/ITO/glass are presented for comparison.

For [C_4_C_1_im][NTf_2_] (Figure 6A), [C_2_C_2_im][NTf_2_] (Figure 6B), and [C_5_C_5_im][NTf_2_] (Figure 6C), a single absorption band is observed at approximately 420 nm in the samples with different thicknesses analyzed, indicating the presence of AgNPs in each sample. These results confirmed the possibility of preparing and stabilizing AgNPs in IL films/coatings and easily evaluating their presence using UV-Vis spectrometry. The thicker the IL film, the better the formation of AgNPs, as shown in Figure 6 (we would like to stress that all these results were obtained using the same amount of Ag deposited on top of each sample). Looking at [C_4_C_1_im][NTf_2_] (Figure 6A), the UV-Vis spectra obtained for the films formed with 200 and 400 ML have a small, broad band that correlates with the lower amount of AgNPs present, and a higher tendency to agglomerate and form a continuous Ag film on top of the IL as suggested by the SEM images (especially Figure 2A2). With thicknesses of 600 and 800 ML, the associated peak becomes more intense, indicating a more significant presence of AgNPs. For this IL, the formation of AgNPs may have reached a point where it will not improve even with thicker IL thin films. At a certain thickness, the height of the IL droplets might be large enough to confine and disperse AgNPs. For [C_2_C_2_im][NTf_2_] (Figure 6B) with 200 ML, there is a less intense band, but it still indicates the presence of AgNPs. For larger film thicknesses (400, 600, and 800 ML), the intensity of the SPR characteristic bands become very similar. This may indicate a large presence of AgNPs on 400, 600, and 800 ML of this IL film. The differentiations observed in the UV-Vis spectra are not so evident in the SEM images, which might indicate a large presence of AgNPs in the inner regions of the IL films. In comparison to the other ILs, the SPR bands associated with each [C_5_C_5_im][NTf_2_]’s film thickness (Figure 6C) show a more gradual increase in intensity and are sharper as the thickness of the thin film increases. These observations can be correlated with the SEM images (Figure 4), where the presence of Ag particles seems to be more pronounced with increasing film thickness. The SPR band of AgNPs in 800 ML of this IL shows the largest amount of these particles among all thin films studied in this work (Figure 6D). Furthermore, as known from the literature, when metal particles increase in size, their absorption peaks shift towards longer wavelengths [66,67]. In comparison to [C_5_C_5_im][NTf_2_], the absorption maxima of [C_2_C_2_im][NTf_2_] and [C_4_C_1_im][NTf_2_] are red-shifted, indicating that the Ag particles are larger in size. In contrast, when analyzing the [C_5_C_5_im][NTf_2_] film, the results strongly suggest that the formation of smaller AgNPs occurred. [C_5_C_5_im][NTf_2_] thus proved to be a very suitable medium for the stabilization of AgNPs. This IL was further investigated by considering the stability of the AgNPs in the IL media and the ability to maintain the stability of the NPs under different conditions. For this purpose, several samples of [C_5_C_5_im][NTf_2_] (800 ML) incorporating AgNPs were subjected to different conditions of air and/or thermal treatment. The UV-Vis spectra for the same Ag/[C_5_C_5_im][NTf_2_] sample at different conditions are presented in Figure 7A: 1—as-deposited AgNPs/[C_5_C_5_im][NTf_2_] film; 2—AgNPs/[C_5_C_5_im][NTf_2_] film exposed to air for 30 min; AgNPs/[C_5_C_5_im][NTf_2_] film thermally treated at 80 °C for 60 min; AgNPs/[C_5_C_5_im][NTf_2_] film thermally treated at 80 °C and further exposed to air for one week. The UV-Vis spectra of an as-deposited AgNPs/[C_2_C_2_im][NTf_2_] film and the same sample exposed to air for one week are presented for comparison (Figure 7B). For all AgNPs/[C_5_C_5_im][NTf_2_] samples (Figure 7A), the SPR band at 420 nm is clearly observed. Remarkably, exposure to air for 30 min did not result in any significant change in terms of the stabilization of AgNPs. Nevertheless, the intensity of the band decreased as the storage conditions were more extreme. A decrease in the intensity of the single band was found for a sample annealed at 80 °C for 60 min. A similar SPR characteristic band was obtained for the same sample exposed to air for one week. The results might indicate a small influence of the storage time in the aggregation of AgNPs. A more noticeable effect is observed with the thermal treatment since an increase in temperature resulted in changes in the film’s viscosity and promoted the diffusion of Ag particles and their subsequent aggregation.

Despite a decrease in its intensity, the SPR band did not disappear, which indicates that the AgNPs were still present in the IL films even after the annealing process. For the congener AgNPs/[C_2_C_2_im][NTf_2_] samples (Figure 7B), the exposure to air for one week resulted in the disappearance of the SPR band. The stability and presence of Ag nanoparticles in this IL medium were found to be time dependent.

**Figure 8:**
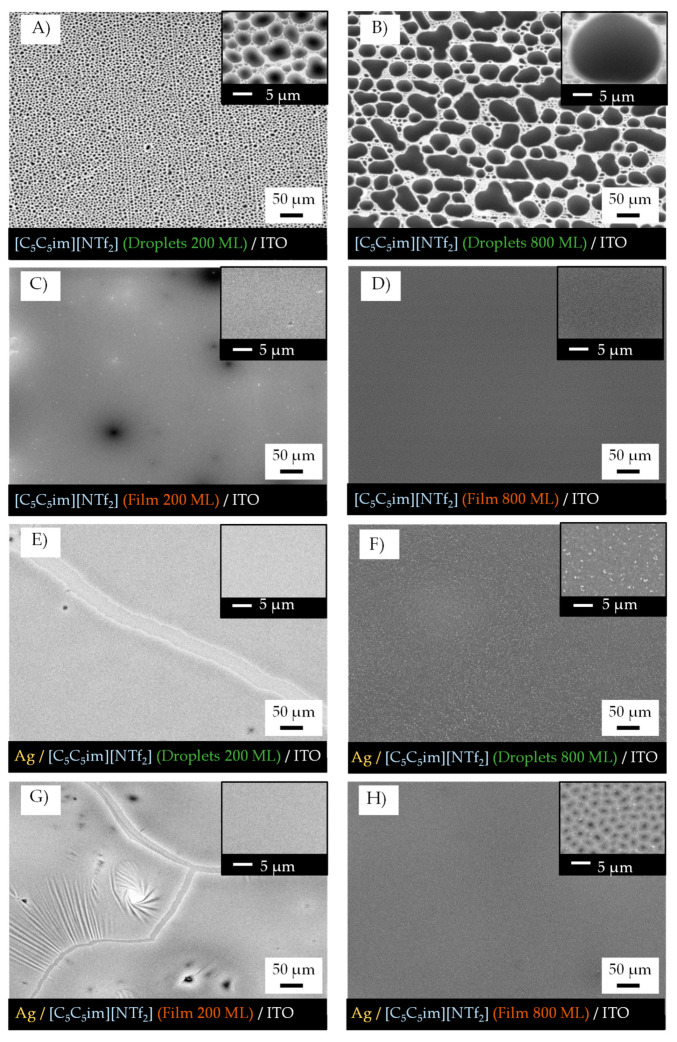
Morphology of as-deposited micro- and nanodroplets of [C_5_C_5_im][NTf_2_] (**A**,**B**) and morphology of coalesced IL films (**C**,**D**). Morphology of the same samples after the deposition of Ag through sputtering for 40 s using a discharge current of 20 mA (**E**–**H**). Micro- and nanodroplets of IL obtained by PVD on ITO/glass surfaces (**A**,**B**) with 200 and 800 ML of thickness. Coalesced IL films were obtained after surface treatment of the IL droplets with argon plasma (no metal target was employed) (**C**,**E**). Samples (**A**,**B**) were exposed to argon plasma and the deposition of AgNPs and the consequent morphologies are presented in (**E**,**F**). Samples (**C**,**D**) were exposed to argon plasma and the deposition of AgNPs and the consequent morphologies are presented in (**G**,**H**). Micrographs acquired through high-resolution SEM using an SE detector. Top views were obtained with a magnification of 500 and 5000× (inset images).

**Figure 9:**
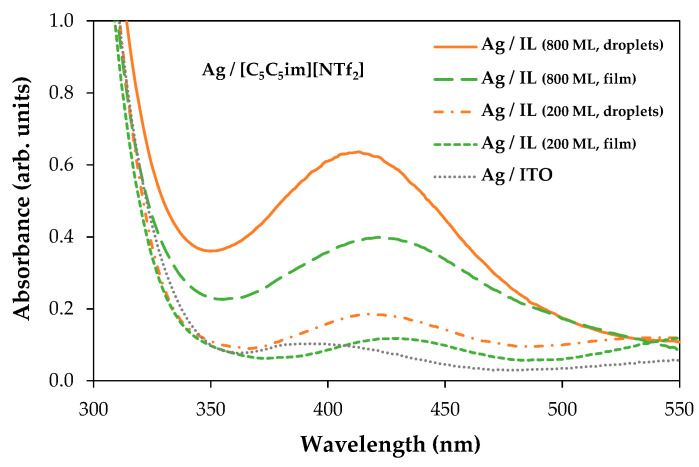
UV-Vis absorption spectra of IL films treated with argon plasma and Ag particles. Spectra for AgNPs/[C_5_C_5_im][NTf_2_] samples using IL droplets or IL coalesced films as the capture medium.

A question can be raised as to what if the IL films had previously coalesced so that they would consist of a continuous thin film rather than droplets as the capture medium for the formation of AgNPs. To explore this effect, [C_5_C_5_im][NTf_2_] was used to make films with thicknesses of 200 ML and 800 ML (Figure 8A,B). The droplets were further treated with argon plasma (no metal target was employed) in order to coalesce and form a continuous film (Figure 8C,D). Afterwards, a sputter deposition of Ag was performed on both samples under the same experimental conditions as described above. The morphology of the samples after exposure to argon plasma and the deposition of AgNPs are presented in Figure 8E,F (the incorporation of AgNPs used microdroplets of IL as capture media) and Figure 8G,H (the incorporation of AgNPs used continuous IL films as capture media). With a plasma surface treatment, the IL droplets coalesced entirely to form a continuous thin film with different thicknesses as seen in Figure 8C (200 ML) and Figure 8D (800 ML). The 800 ML film surface seemed to be more homogenous. When the less-thick samples (200 ML) were exposed to the deposition of AgNPs, the formation of a Ag thin film on the surface of the IL could be observed (Figure 8E,G). This result occurred for both capture media (microdroplets or coalesced film). On the contrary, the 800 ML films were capable of forming and stabilizing the Ag nanoparticles while avoiding their aggregation, especially for the Ag deposited using microdroplets of IL as the capture medium (Figure 8F). On the 200 ML film (Figure 8G) there was a formation of slits and wrinkles at the surface while on the 800 ML film (Figure 8H) the surface was more homogenous with black spots, which indicated the presence of agglomerates of IL surrounded by silver resembling a protective layer dividing the IL into different portions. Detailed images of the wrinkled structures are presented in Figures S10 and S11. Both samples were analyzed using UV-Vis spectroscopy to verify the presence of AgNPs (Figure 9). Every sample revealed unique SPR absorption bands in the region of 400–420 nm that corresponded to the presence of AgNPs. The thin films with 200 ML had a lower AgNPs content compared to the films with 800 ML, as expected and discussed before. However, the samples with the same thickness showed differences between them. A more intense and broader band could be observed when the AgNPs had been incorporated using microdroplets of IL as the capture medium. Moreover, the absorption peak of the AgNPs deposited on a coalesced IL was observed to red shift, which is likely due to the presence of larger AgNPs [66,67]. These observations reveal that the presence of droplets as a confining medium helps in the formation, dispersion, and stabilization of the NPs.

## 3. Discussion

The results reported in this work highlight the potential applications of different imidazolium-based IL films as capture media for the formation of AgNPs. The use of different cation alkyl side chain lengths and symmetries was a strategy used to understand how the size of the alkyl chains may affect the stabilization of AgNPs. It has been reported that in the bulk state, the ILs with longer side chains reveal a tendency towards the segregation of MNPs that does not result in particle agglomeration. We can, to some extent, correlate our results with the available information for the bulk ILs [47,68]. Considering the three ILs studied, the [C_5_C_5_im][NTf_2_] film provides a more suitable medium for the formation and dispersion of AgNPs. The vacuum-deposition process of this IL on ITO surfaces led to the formation of large droplets as shown in Figure 4. These droplets behaved as confining sites for the stabilization of NPs. The alkyl side chains of this IL might play a relevant role in the stabilization of AgNPs. In fact, ILs composed of larger cation alkyl side chain lengths are reported to favor the formation of AgNPs with lower diameters and more uniform size distribution [47,68]. The alkyl chains of the IL cations have a strong effect on the steric stabilization of NPs. When the cationic moieties constituted by long alkyl side chains are close to the nanoparticle surface, they provide steric forces by stretching out their bulky side chains, thus hindering the MNPs from moving towards each other and aggregating [68]. The segregation of AgNPs could then be highly dependent on the arrangement of the alkyl side chains in the imidazolium-based ILs. For shorter alkyl side chains, such as in [C_2_C_2_im], the particle aggregation seems to be more favorable. A complete aggregation results in the formation of a Ag film on the surface of the IL as revealed by the SEM and UV-Vis analyses (Figure 3 and Figure 6).

This work also demonstrated the significant impact of the film’s thickness on the stabilization of AgNPs, since most NPs are stabilized in the inner regions of the IL film, especially within the precursor IL droplets. At smaller IL film thicknesses, some considerations are critical, such as the size of the droplets and the distance between the ITO substrate and the surface of the IL. The sputtering process did not only induce the deposition of metal particles but also the coalescence of the IL droplets due to the effect of the argon plasma. Hence, the deposited silver is somehow dragged along by the droplets that are moving at the beginning of the sputtering process, prior to total thin-film coalescence. When the IL film is formed by a high surface density of micro- and nanodroplets with a small size, the concomitance of the plasma surface treatment and the Ag deposition may lead to a more chaotic distribution of the AgNPs on the surface of the IL and at the border of the precursor droplets, resulting in a higher propensity for agglomeration and the formation of a thin Ag film at the IL surface. Note that the wrinkled structures observed and shown in Figure 8G are typical for metal films deposited on soft surfaces [69,70,71,72]. Furthermore, if the substrate has a thinner IL layer, for example with 200 ML, the Ag particles penetrate this layer more easily and can hit the ITO substrate. These reasons could explain why the stabilization of AgNPs in very thin IL films does not work as well as in thicker ones, since for these latter samples the height of the IL droplets is significantly larger and capable of confining the AgNPs in their inner regions. The stabilization of AgNPs depends not only on the intrinsic chemical features of the ILs and their ability to stabilize NPs, but also on the movement of the IL droplets due to the surface treatment. The argon plasma treatment has a more preponderant effect on the movement and consequent coalescence of the smaller droplets. The large droplets encapsulate the sputtered particles and decrease their agglomeration at the surface, resulting in the segregation and better distribution of AgNPs. In fact, we obtained better results in the formation of AgNPs when ≥600 ML films were employed. The UV-Vis’s characterization confirmed the formation of AgNPs by a sputter deposition of Ag on IL films, and the results are consistent with those reported in the literature for AgNPs synthesized by other methods [66,67]. Regarding the differences in ILs, [C_5_C_5_im][NTf_2_] proved to be the best in terms of the stabilization of AgNPs. With thicker films, the ideal conditions for the best performance become clear: (i) big droplets to encapsulate the AgNPs; (ii) larger minimal areas of nucleation which lead to bigger IL droplets being formed and fewer nanodroplets alongside the borders of the bigger ones; and (iii) higher numbers of carbons on the alkyl side chains which lead to the segregation of NPs and prevent the formation of clusters and/or Ag films on the surface. Indeed, different conditions such as a change in the temperature of the capture medium can affect the size and size distribution of MNPs during synthesis [73,74]. The IL films composed of micro- and nanodroplets are very stable, either in contact with air or at high temperatures [21]. Nevertheless, air exposure and thermal annealing can highly influence the aggregation of NPs when incorporated into a two-dimensional film of IL. This observation was here clearly shown for the [C_2_C_2_im][NTf_2_] sample. Noticeably, for [C_5_C_5_im][NTf_2_], only a small effect of air exposure was observed. For this IL, the AgNPs show a quite high time stability. Exposure to ambient conditions might not disturb the stabilization of the AgNPs, and their properties might remain intact. This result is probably derived from the steric stabilization of the NPs induced by the alkyl side chains of the [C_5_C_5_im][NTf_2_] sample. In addition, the dispersion and segregation of AgNPs into this IL, using a capture medium composed of large droplets, favor the dispersion and segregation of the Ag and reduce the formation of a thin Ag film on the surface.

## 4. Materials and Methods

### 4.1. Ionic Liquids

The ILs 1-butyl-3-methylimidazolium bis(trifluoromethanesulfonyl)imide ([C_4_C_1_im][NTf_2_]), 1,3-diethyl-imidazolium bis(trifluoromethanesulfonyl)imide ([C_2_C_2_im][NTf_2_]), and 1,3-dipenthyl-imidazolium bis(trifluoromethanesulfonyl)imide ([C_5_C_5_im][NTf_2_]) were purchased from IoLiTec with a state purity of >99%. The volatile content of [C_4_C_1_im][NTf_2_], [C_2_C_2_im][NTf_2_], and [C_5_C_5_im][NTf_2_] was removed by vacuum thermal evaporation (*p* < 10^−4^ Pa) at *T* = 423 K, prior to thin-film deposition. The molecular structures of the IL cation–anion pairs are depicted in Figure 1A.

### 4.2. Vacuum Thermal Evaporation of Ionic Liquids

Micro- and nanodroplets of [C_4_C_1_im][NTf_2_], [C_2_C_2_im][NTf_2_], and [C_5_C_5_im][NTf_2_] were obtained from glass substrates coated with indium tin oxide (ITO, ~180 nm of thickness). ITO/glass substrates (10 mm × 10 mm × 1.1 mm) were purchased commercially from Praezisions Glas & Optik GmbH. These substrates were cleaned with high-purity ethanol in an ultrasonic bath and dried with ultra high purity grade argon (>99.9%). The IL thin films were prepared using a customized procedure of physical vapor deposition (PVD, details presented in Figures S1–S3) based on the Knudsen effusion method (ThinFilmVD) (Figure 1B) [56]. With the use of Knudsen effusion cells, it was possible to obtain very accurate control of the mass flow rate (*Φ*). *Φ* was determined according to Equation (1), where *Φ*s and *Φ*kc represent the mass flow rate on the substrate surface and from the orifice of the Knudsen cell, respectively [21,22,23,24,48,75]. The real-time evaluation and control of *Φ*s were performed using a 6 MHz gold-coated quartz crystal microbalance (QCM). The relation between *Φ*s and *Φ*kc depends on a geometric factor *g*, which is affected by the distance between the evaporation source and the substrate surface. *Φ*kc is derived from the Knudsen effusion technique (Equation (1)), where *T* represents the effusion temperature of the sample, *p* is the vapor pressure at *T*, *w*_o_ the transmission probability factor, *m* is the mass of the effused vapor, and *M* its molar mass, *t* is the time of deposition, and *A*_o_ the area of the Knudsen cell orifice [75]. The fabrication of IL films (micro- and nanodroplets) was made on ITO/glass slides using a substrate support system, kept at a constant temperature *T* = 283 K. IL films with different thicknesses (200, 400, 600, and 800 ML) were fabricated under similar experimental conditions (mass flow rates on the substrate surface (*Φ*s) between 0.05 and 0.10 nm∙s^−1^ were applied). [C_4_C_1_im][NTf_2_], [C_2_C_2_im][NTf_2_], and [C_5_C_5_im][NTf_2_] were evaporated at *T* = 483 K, *T* = 488 K, and *T* = 488 K, respectively. More details are presented in Table S1.

(1) $$\Phi_{s}=g\;\cdot \Phi_{\mathrm{kc}}=g\cdot \frac{p\;\cdot \;w_{o}\cdot \sqrt{M}}{\sqrt{2\pi RT}}=g\cdot \frac{m}{A_{o}\cdot t}$$

This PVD method ensures the preparation of very well defined IL films with precisely known thicknesses and high reproducibility [21,22,23,24,48,73]. To ensure the purity of the ILs, the pressure in the chamber was lowered to ~10^−4^ Pa and the oven temperature was monitored at 423 K for ~30 min before the deposition of each IL. This process allowed an in situ purification of each IL. Since the deposition of ILs by PVD produces thin films constituted of droplets, the thickness of these films is usually given by monolayers (ML), where 1 ML is equivalent to one closed layer of ion pairs with cations and anions on top of each other. Equation (2) can be used to estimate the height (*h*) of 1 ML [76]:

(2) $$h=\sqrt[{3}]{\frac{V_{m}}{N_{A}}}=\sqrt[{3}]{\frac{M}{N_{A}\times \rho }}$$

where *M* is the molar mass of the IL, *V*_m_ is the bulk molecular volume, *ρ* the mass density, and *N*_A_ is the Avogadro constant. Using this equation we can predict that thicknesses of *h* = 7.9 Å, *h* = 7.7 Å, and *h* = 8.6 Å correspond to 1 ML of [C_4_C_1_im][NTf_2_] (*M* = 419.4 g.mol^−1^ and *ρ* = 1.44 g.cm^−3^ at 283.2 K), [C_2_C_2_im][NTf_2_] (*M* = 405.3 g.mol^−1^ and *ρ* = 1.49 g.cm^−3^ at 283.2 K), and [C_5_C_5_im][NTf_2_] (*M* = 489.5 g.mol^−1^ and *ρ* = 1.31 g.cm^−3^ at 283.2 K), respectively [77]. Thin films of 200 ML (*h* ≈ 157 nm, *h* ≈ 154 nm, and *h* ≈ 171 nm for [C_4_C_1_im], [C_2_C_2_im], and [C_5_C_5_im], respectively), 400 ML (*h* ≈ 314 nm, *h* ≈ 308 nm, and *h* ≈ 343 nm for [C_4_C_1_im], [C_2_C_2_im], and [C_5_C_5_im], respectively), 600 ML (*h* ≈ 471 nm, *h* ≈ 461 nm, and *h* ≈ 514 nm for [C_4_C_1_im], [C_2_C_2_im], and [C_5_C_5_im], respectively) and 800 ML (*h* ≈ 628 nm, *h* ≈ 615 nm, and *h* ≈ 686 nm for [C_4_C_1_im], [C_2_C_2_im], and [C_5_C_5_im], respectively) were obtained in order to infer the effects of the film’s thickness on the formation and stabilization of AgNPs.

### 4.3. Sputter Deposition of Silver Particles in ILs

Ag particles were deposited on the surface of IL films by pulsed DC magnetron sputtering using the Cressington 108 Auto Sputter Coater instrument. A high-purity (>99.9%) Ag target was employed. Argon plasma treatment at low pressure (<10 Pa) was used simultaneously with the Ag deposition, from the moment the deposition starts until its end, with a discharge current of 20 mA (Figure 1C). A constant deposition time of 40 s was used in all experiments. In these experimental conditions, the QCM sensor indicated an overall Ag thickness of ≈13 nm. Due to the surface treatment with argon plasma, the coalescence of micro- and nanodroplets of the ILs to form continuous thin films was also promoted.

### 4.4. High-Resolution Scanning Electron Microscopy

The morphology and topography of the IL films, before and after the deposition of AgNPs, were studied using scanning electron microscopy (SEM) through the high-resolution FEI Quanta 400 FEG ESEM/EDAX Genesis X4M instrument at the CEMUP (Centro de Materiais da Universidade do Porto) services. Two different detectors, a secondary electron detector (SE) and a backscattered electron detector (BSE), were employed and micrographs were obtained at different magnifications—500×, 2000×, and 5000×. A better analysis of the region closer to the surface of the film was used with the SE detector, where topographical images with high resolution were obtained, allowing us to observe the contrast, texture, and rugosity of the thin film. For inner regions of the film, the BSE detector allowed a compositional and topographical study of the films, but with less resolution than the SE. These two methods allowed the observation and quantification of the size and shape of the micro- and nanodroplets formed in each IL film before Ag deposition. After metal deposition, it was possible to observe that the ILs micro- and nanodroplets had coalesced, forming a continuous thin film. In this film, the dispersion and/or aggregation of Ag nanoparticles was observed, near the surface as well as in the inner regions. The SEM characterization was carried out 1 or 2 days after sample preparation and storage. The samples were stored in a desiccator to avoid moisture contamination.

### 4.5. UV-Vis Spectroscopy

UV-Vis characterization of all IL films with different thicknesses after the deposition of AgNPs was performed with a diode array spectrophotometer using an Agilent 8453 UV-Vis spectroscopy system. The experimental spectra were obtained in the range of 200–800 nm at 298 K. This method was used to confirm the presence of AgNPs in the coalesced IL films, by inferring the characteristic surface plasmon resonance (SPR) peak. The presence of an intense band in the region of 400–420 nm indicated the presence of AgNPs in the IL films/coatings.

## 5. Conclusions

This work describes the formation and stabilization of silver nanoparticles (AgNPs) through sputtering onto thin ionic liquid (IL) films composed of microdroplets or two-dimensional coalesced films. Three ionic liquids were used to unveil the characteristics of the dispersion and stabilization of AgNPs into different IL capture media ([C_2_C_2_im][NTf_2_], [C_4_C_1_im][NTf_2_], and [C_5_C_5_im][NTf_2_]). The dispersion of nanoparticles was found to be significantly affected by the thickness of the ILs’ films, and larger film thicknesses were found to be the optimal capture media for the formation and stabilization of AgNPs. The UV-Vis spectrum of the samples indicated the presence of AgNPs in the inner regions of the thicker IL films, while SEM characterization showed a greater tendency towards the formation of a thin silver film on the surface of the thinner IL films. Notably, the [C_5_C_5_im][NTf_2_] films exhibited promising results, demonstrating a controlled deposition of AgNPs in the ILs’ thin films. Specifically, PVD of this IL resulted in the formation of films consisting of large and stable droplets that acted as a confining medium for the segregation and stabilization of the AgNPs.

## Data Availability

Not applicable.

## Supplementary Materials

The following supporting information can be downloaded at: https://www.mdpi.com/article/10.3390/molecules28073029/s1, Figure S1: Schematic representation of the vapor deposition/vacuum thermal evaporation methodology; Figure S2: Schematic representation of the ovens of the ThinFilmVD apparatus; Figure S3: Schematic representation and images of the substrate support system; Figure S4: Illustration of the typical mechanisms of nucleation and growth of ionic liquid micro- and nanodroplets; Figure S5: Morphology of the substrate (ITO/glass); Figure S6: Micrographs of ITO-coated glass substrates treated with argon plasma and Ag particles; Figure S7: Polarized light microscopy images of ionic liquid films treated with argon plasma and Ag particles; Figure S8: Micrographs of ionic liquid films treated with argon plasma and Ag particles. Morphology of [C_4_C_1_im][NTf_2_] and [C_5_C_5_im][NTf_2_] obtained using both a secondary electron detector and a backscattered electron detector; Figure S9: Detailed morphology of the surfaces of [C_4_C_1_im][NTf_2_] and [C_5_C_5_im][NTf_2_] films analyzed after surface treatment with argon plasma and deposition of Ag. Graphs/histograms presenting the size distribution of AgNPs on the surfaces of the films; Figure S10: Micrographs of ionic liquid films treated with argon plasma and Ag particles. Morphology for 200 ML of [C_2_C_2_im][NTf_2_], [C_4_C_1_im][NTf_2_], and [C_5_C_5_im][NTf_2_]; Figure S11: Micrographs of ionic liquid films treated with argon plasma and Ag particles. Detailed morphology for 200 ML of [C_5_C_5_im][NTf_2_] evidencing the presence of a thin film of Ag onto the 200 ML film; Table S1: Experimental conditions for the physical vapor deposition of each ionic liquid.
