# Supplementary material for: Confined Silver Nanoparticles in Ionic Liquid Films
Source: Molecules. 2023 Mar 28;28(7):3029. doi: 10.3390/molecules28073029 (PMC10095659; doi:10.3390/molecules28073029)
Supplement: Supplementary file 1 [file molecules-28-03029-s001.zip › molecules-2294392-supplementary.pdf]

Supporting Information

Confined Silver Nanoparticles in Ionic Liquid Films

Alexandre C. P. M. Alves, Luís M. N. B. F. Santos, Margarida Bastos, and José C. S. Costa *

CIQUP, Institute of Molecular Sciences (IMS), Department of Chemistry and Biochemistry, Faculty of Science, University of Porto, Rua do Campo Alegre, P4169 007 Porto, Portugal
** Correspondence: jose.costa@fc.up.pt*

Supplementary Materials

Figure S1: Schematic representation of the vapor deposition/vacuum thermal evaporation methodology; Figure S2: Schematic representation of the ovens of the ThinFilmVD apparatus; Figure S3: Schematic representation and images of the substrate support system; Figure S4: Illustration of the typical mechanisms of nucleation and growth of ionic liquid micro- and nanodroplets; Figure S5: Morphology of the substrate (ITO/glass); Figure S6: Micrographs of ITO-coated glass substrates treated with argon plasma and Ag particles; Figure S7: Polarized light microscopy images of ionic liquid films treated with argon plasma and Ag particles; Figure S8: Micrographs of ionic liquid films treated with argon plasma and Ag particles. Morphology of [C_4C_1im][NTf_2] and [C_5C_5im][NTf_2] obtained using both a secondary electron detector and a backscattered electron detector; Figure S9: Detailed morphology of the surfaces of [C_4C_1im][NTf_2] and [C_5C_5im][NTf_2] films analyzed after surface treatment with argon plasma and deposition of Ag. Graphs/histograms presenting the size distribution of AgNPs on the surfaces of the films.; Figure S10: Micrographs of ionic liquid films treated with argon plasma and Ag particles. Morphology for 200 ML of [C_2C_2im][NTf_2], [C_4C_1im][NTf_2], and [C_5C_5im][NTf_2]; Figure S11: Micrographs of ionic liquid films treated with argon plasma and Ag particles. Detailed morphology for 200 ML of [C_5C_5im][NTf_2] evidencing the presence of a thin film of Ag onto the 200 ML film; Table S1: Experimental conditions for the physical vapor deposition of each ionic liquid.

Index

**Figure S1**. Schematic representation of the vapor deposition/vacuum thermal evaporation methodology.	**S3**
**Figure S2**. Schematic representation of the ovens of the ThinFilmVD apparatus.	**S3**
**Figure S3**. Schematic representation and images of the substrate support system.	**S4**
**Figure S4**. Illustration of the typical mechanisms of nucleation and growth of ionic liquid micro- and nanodroplets.	**S4**
**Figure S5.** Morphology of the substrate (ITO/glass).	**S5**
**Figure S6**. Micrographs of ITO-coated glass substrates treated with argon plasma and Ag particles.	**S5**
**Figure S7**. Polarized light microscopy images of ionic liquid films treated with argon plasma and Ag particles.	**S6**
**Figure S8**. Micrographs of ionic liquid films treated with argon plasma and Ag particles. Morphology of [C_4C_1im][NTf_2] and [C_5C_5im][NTf_2] obtained using both a secondary electron detector and a backscattered electron detector.	**S7**
**Figure S9**. Detailed morphology of the surfaces of [C_4C_1im][NTf_2] and [C_5C_5im][NTf_2] films analyzed after surface treatment with argon plasma and deposition of Ag. Graphs/histograms presenting the size distribution of AgNPs on the surfaces of the films.	**S8**
**Figure S10**. Micrographs of ionic liquid films treated with argon plasma and Ag particles. Morphology for 200 ML of [C_2C_2im][NTf_2], [C_4C_1im][NTf_2], and [C_5C_5im][NTf_2].	**S9**
**Figure S11**. Micrographs of ionic liquid films treated with argon plasma and Ag particles. Detailed morphology for 200 ML of [C_5C_5im][NTf_2] evidencing the presence of a thin film of Ag onto the 200 ML film.	**S10**
**Table S1**. Experimental conditions for the physical vapor deposition of each ionic liquid.	**S11**

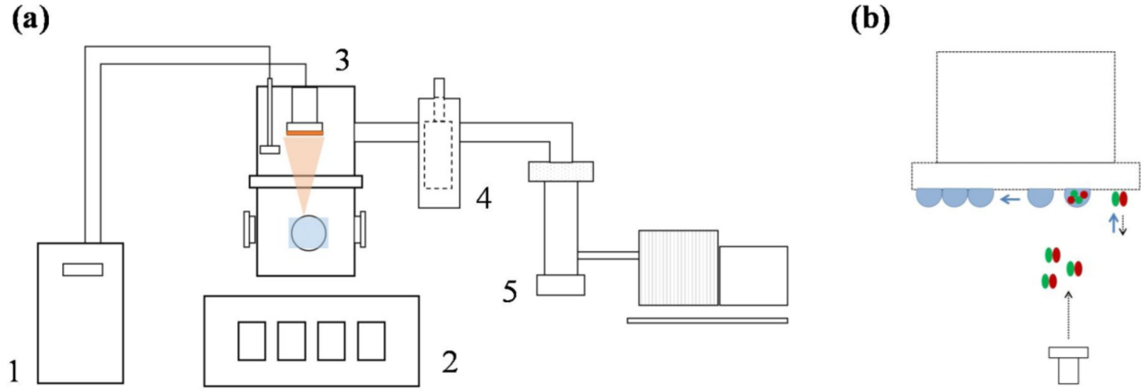

Figure S1. Schematic representation of the vapor deposition/vacuum thermal evaporation methodology: (a) ThinFilmVD apparatus (1 – cooling system, 2 – instrumentation box, 3 – vacuum chamber, 4 – N_2 (l) metallic trap, 5 – vacuum pumping system); (b) schematic detail of the PVD of ionic liquids by vacuum thermal evaporation from a Knudsen cell. More details: *Appl. Surf. Sci.*, 2018, 428, 242 and *J. Chem. Eng. Data*, 2015, 60, 3776.

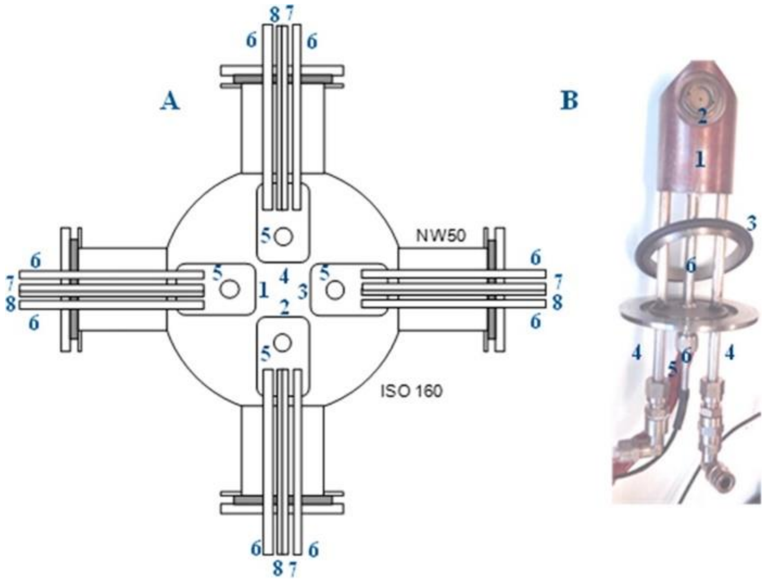

Figure S2. A – Schematic representation of the ovens of the ThinFilmVD apparatus: 1, 2, 3, 4 – individual copper ovens; 5 – cavity of the Knudsen cell screwing; 6 – air cooling tube; 7 – heater; 8, – Pt100 sensor; B – Image of an individual oven (top view): 1 – copper block; 2 – Knudsen cell; 3 – Viton O-ring; 4 – cooling system; 5 – heater; 6 – Pt100. More details: *J. Chem. Eng. Data*, 2015, 60, 3776.

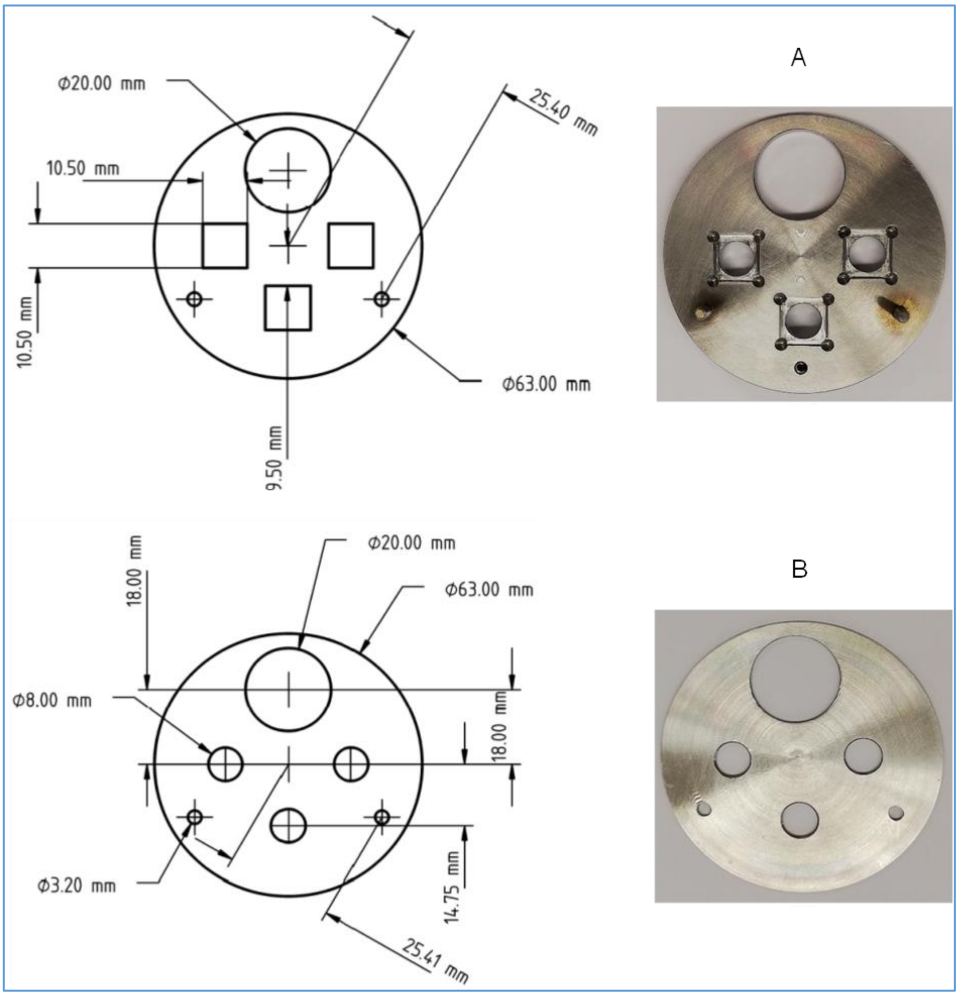

Figure S3. Schematic representation (left) and images (right) of the substrates support system. The support was used for the deposition of each ionic liquid (up to 3 film samples) on ITO/glass surfaces.

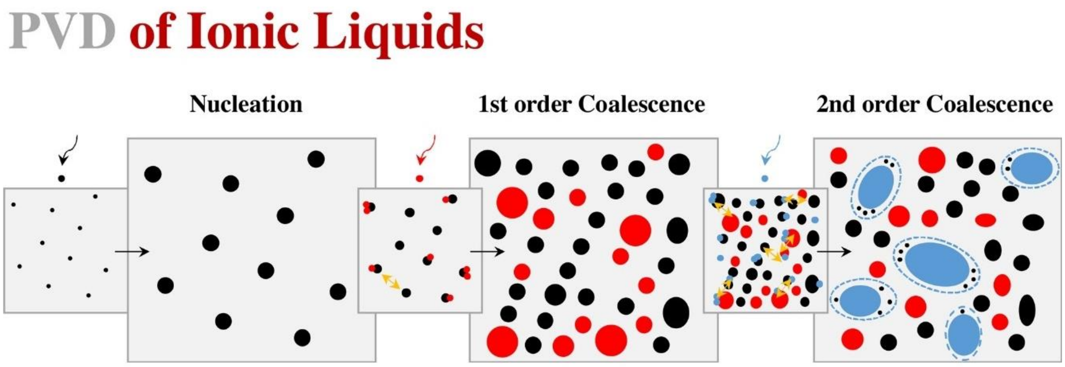

Figure S4. Illustration of the typical mechanisms of nucleation and growth of ionic liquid micro- and nanodroplets (precursor ionic liquid films): minimum free area to promote nucleation (MFAN); first-order coalescence; second-order coalescence. More details: *Appl. Surf. Sci.*, 2018, 428, 242.

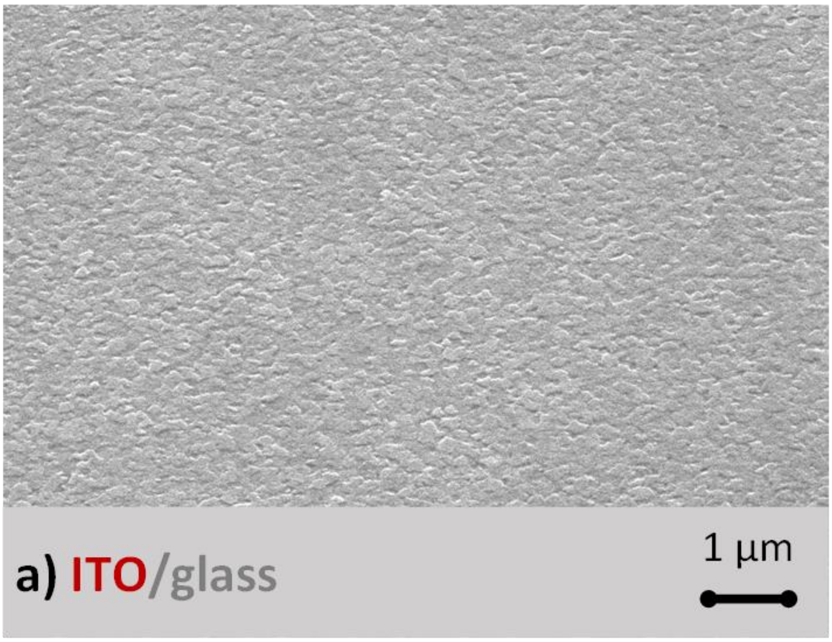

Figure S5. Detailed morphology of the indium tin oxide (ITO)/glass surface. Micrograph acquired through high-resolution scanning electron microscopy (SEM) by using a secondary electron detector (SED). A lateral view at 45° was obtained with a magnification of 25000×.

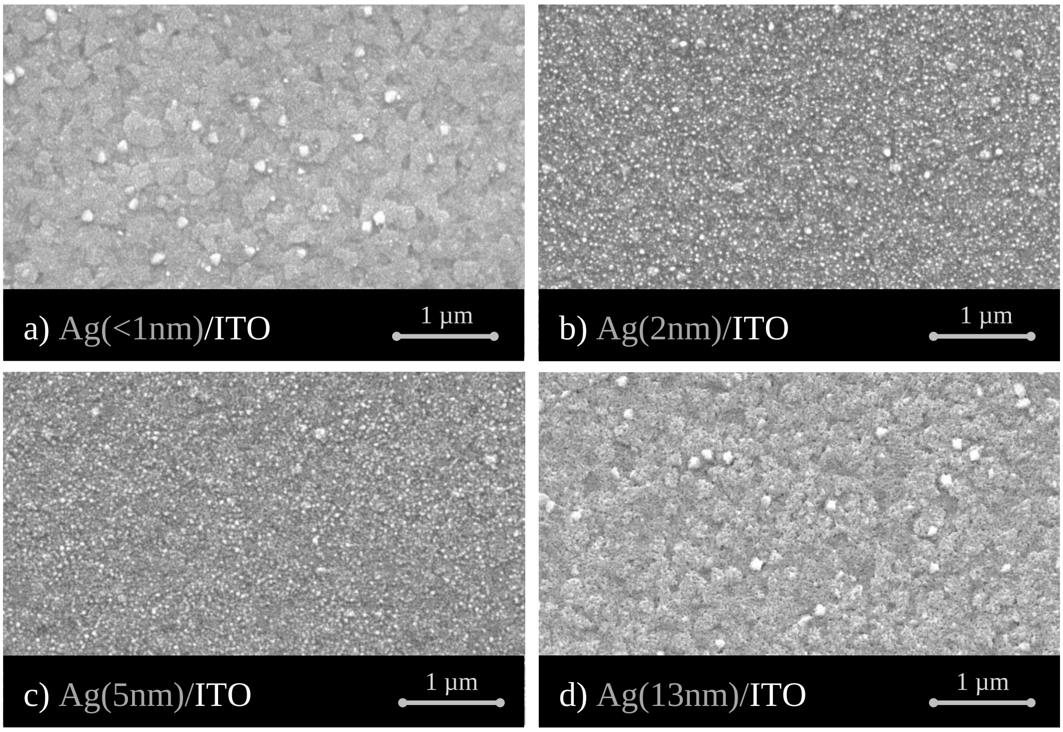

Figure S6. Micrographs of ITO-coated glass substrates treated with argon plasma and silver (Ag) particles. Morphology of the ITO surface after 5 (1 nm, image a), 10 (2 nm, image b), 20 (5 nm, image c), and 40 (13 nm, image d) s of sputter deposition from a silver magnetron target and argon plasma using a discharge current of 20 mA. Top views (50 000 ×) were acquired through high-resolution scanning electron microscopy (SEM) by using a secondary electrons detector (SED).
For lower amounts of Ag (2 or 5 nm, images b, and c), the formation of individual Ag clusters onto ITO can be observed. The increase of the Ag thickness (13 nm, image d) leads to the coalescence of the clusters and a formation of a thin film of Ag onto the substrate.

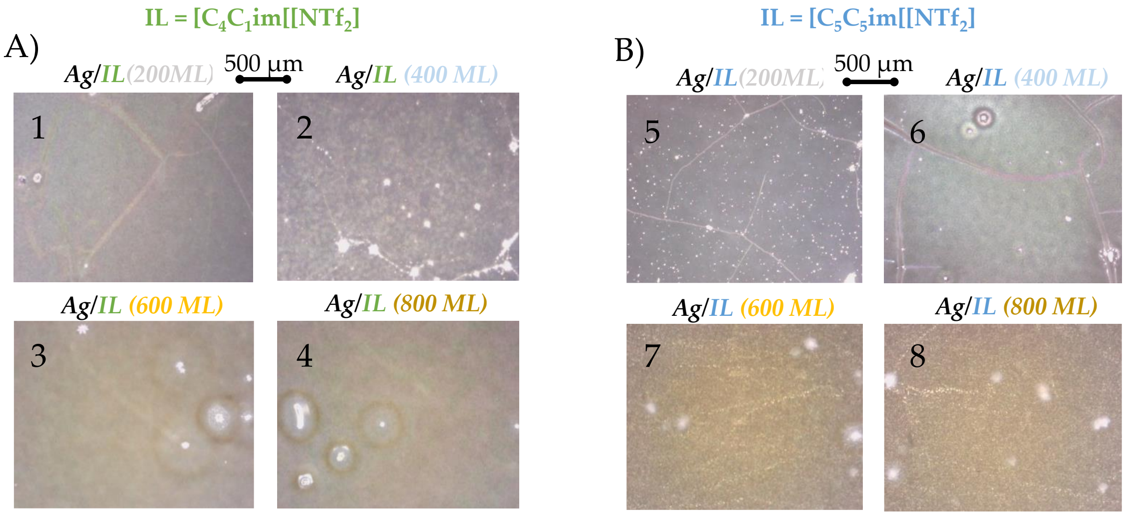

Figure S7. Polarized light microscopy images (top views, 150 ×) of ionic liquid films treated with argon plasma and silver (Ag) particles. Micrographs for 200, 400, 600, and 800 ML of $[C_4C_1im][NTf_2]$ (A, 1, 2, 3, 4), and $[C_5C_5im][NTf_2]$ (B, 5, 6, 7, 8). Micro- and nanodroplets of ionic liquids previously prepared by vacuum thermal evaporation onto ITO-coated glass substrates. Ag deposition was performed through 40 s (13 nm) of sputter deposition from a silver magnetron target and argon plasma (inducing the droplet coalescence process) using a discharge current of 20 mA.
Wrinkle structures are depicted for Ag deposited onto the thinner IL films (200 and 400 ML, images 1, 2, 5, 6) highlighting the formation of an Ag film on the surface of the IL films. For thicker IL films (600 and 800 ML, images 3, 4, 7, 8), the images appear yellow (characteristic colour of the AgNPs) indicating the formation/stabilization of AgNPs into the inner regions of the IL film.

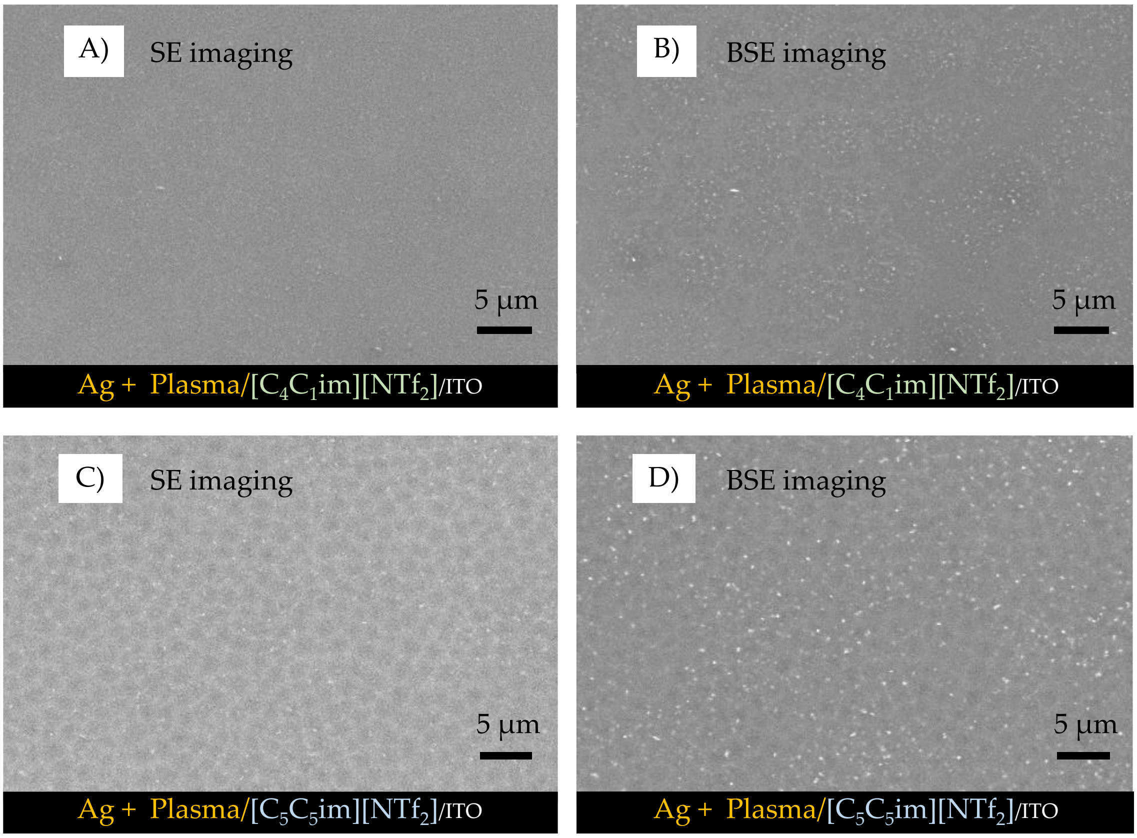

Figure S8. Micrographs of ionic liquid films treated with argon plasma and Ag particles. Morphology for 600 ML of [C_4C_1im][NTf_2] (A and B) and [C_5C_5im][NTf_2] (C and D) obtained using both a secondary electron detector (A and C) and a backscattered electron detector (B and D). Upon comparing both images, it can be strongly inferred that the white spots are corresponding to the AgNPs.

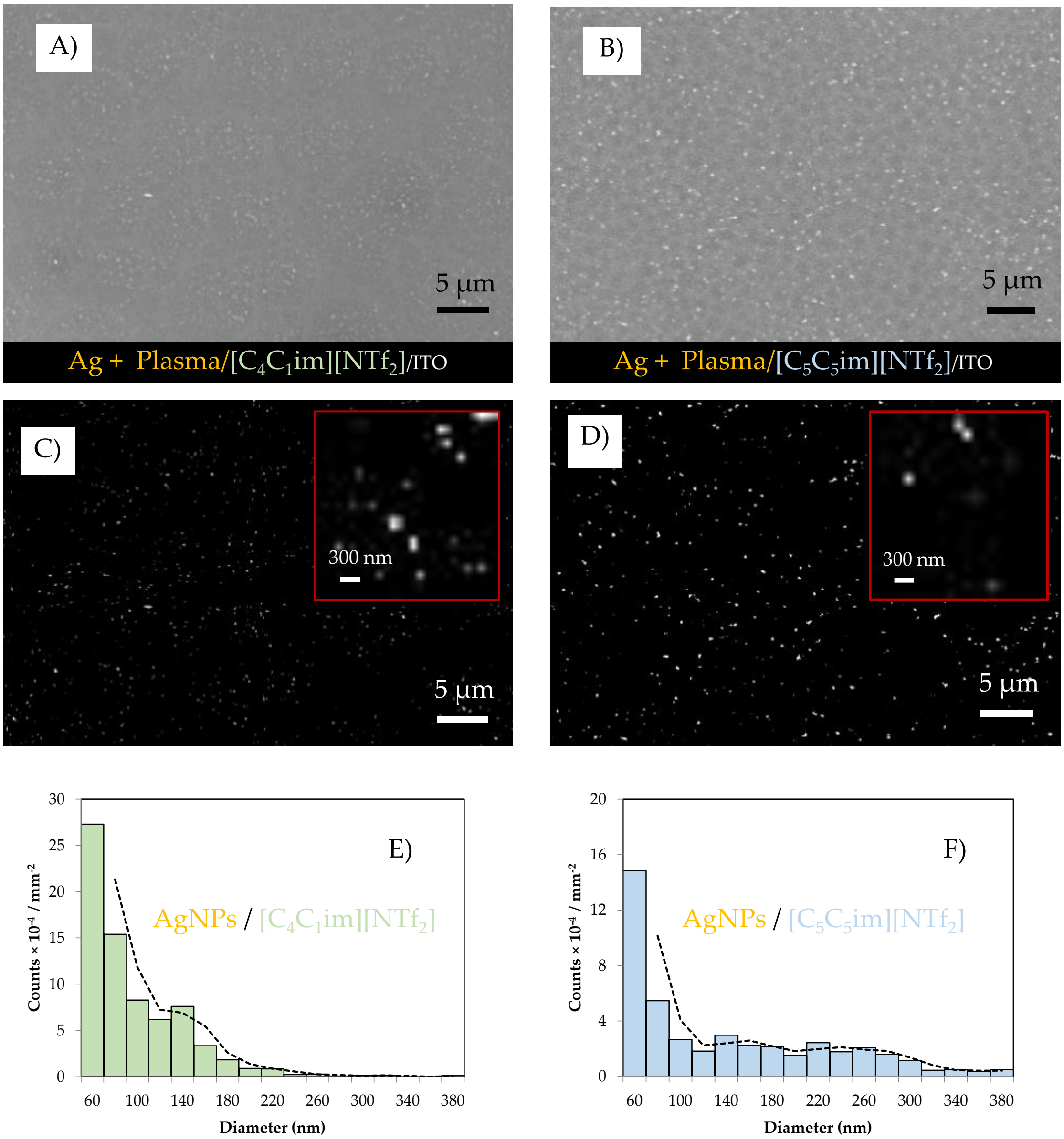

Figure S9. Detailed morphology (micrographs acquired through high-resolution SEM using the BSE detector) of the surfaces of [C_4C_1im][NTf_2] (A) and [C_5C_5im][NTf_2] (B) films (600 ML) analyzed after surface treatment with argon plasma and deposition of Ag. Images C and D show the presence of AgNPs in the IL films. These images were obtained by processing SEM micrographs using ImageJ software. Graphs/histograms E and F present the size distribution of AgNPs on the surfaces of [C_4C_1im][NTf_2] and [C_5C_5im][NTf_2], respectively.

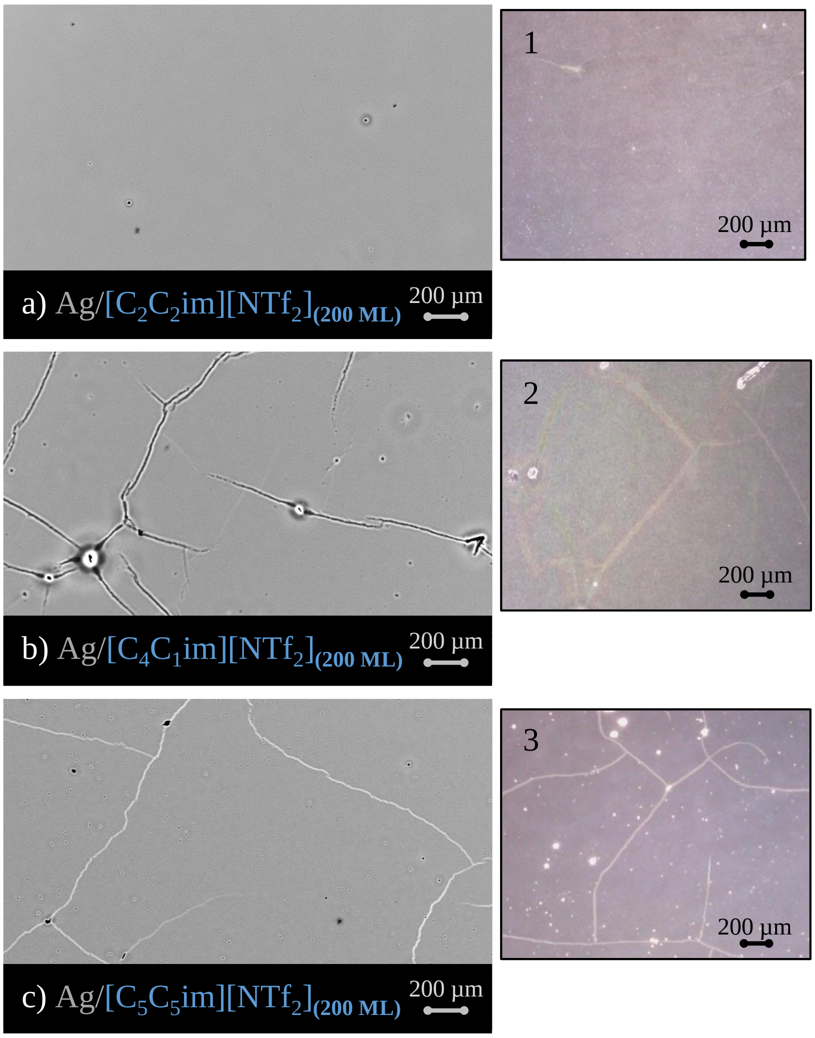

Figure S10. Micrographs of ionic liquid films treated with argon plasma and silver (Ag) particles. Morphology for 200 ML of [C_2C_2im][NTf_2] (a, 1), [C_4C_1im][NTf_2] (b, 2), and [C_5C_5im][NTf_2] (c, 3). Ionic liquid films prepared by a droplet coalescence process induced by argon plasma treatment; micro- and nanodroplets of ionic liquids previously prepared by vacuum thermal evaporation onto ITO-coated glass substrates. Ag deposition was performed through 40 s (13 nm) of sputter deposition from a silver magnetron target and argon plasma using a discharge current of 20 mA. Top views were acquired through high-resolution scanning electron microscopy (SEM) (images a, b, c), by using a backscattered electron detector (BSED), and through polarized light microscopy (images 1, 2, 3).

The SEM images reveal the formation of some crack patterns on the layer surface, which is a strong indication of the aggregation of Ag particles and the consequent formation of a thin metallic film onto the IL surface.

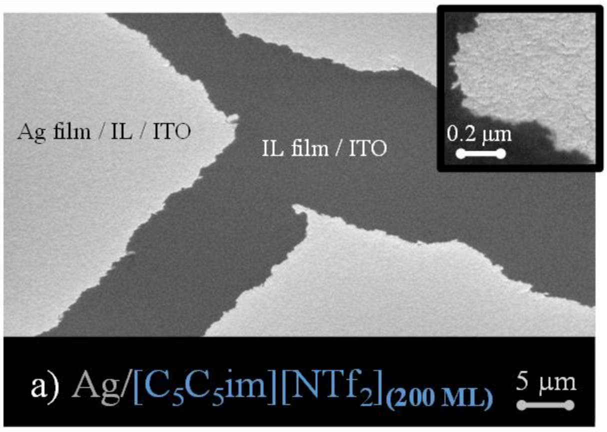

Figure S11. Micrographs of ionic liquid films treated with argon plasma and silver (Ag) particles. Detailed morphology for 200 ML of [C_5C_5im][NTf_2] evidencing the presence of a thin film of Ag onto the 200 ML film. Ionic liquid film prepared by a droplet coalescence process induced by argon plasma treatment; micro- and nanodroplets of ionic liquid previously prepared by vacuum thermal evaporation onto ITO-coated glass substrates. Ag deposition was performed through 40 s (13 nm) of sputter deposition from a silver magnetron target and argon plasma using a discharge current of 20 mA. Top views were acquired through high-resolution scanning electron microscopy (SEM) by using a secondary electron detector (SED).
The image depicts the fracture of the thin Ag film formed on the IL surface.

Table S1. Experimental conditions for the physical vapor deposition/thermal evaporation of each ionic liquid: effusion temperature ($T_{eff.}$); equilibrium vapor pressure (*EVP*); mass flow rate at the Knudsen effusion cell orifice (Φ (Knudsen cell)); mass flow rate at the substrate surface (Φ (QCM)) and corresponding deposition rate in Å·s^{-1}; geometric factor; deposition time; thin film thickness (nm and ML).

Precursor	$T_{eff.}$ K	*EVP* Pa	Φ (Knudsen cell) µg·cm^{-2}·s^{-1}	Φ (QCM) ng·cm^{-2}·s^{-1}	Geometric factor	Deposition rate Å·s^{-1}	Deposition time min	Thickness ML
				**[C_2C_2im][NTf_2] (200, 400, 600, 800 ML) / ITO-glass**				
**[C_2C_2im][NTf_2]**	488	≈ 0.16	≈ 58	11.8 ± 1.5	≈ 2×10^{-4}	0.8 ± 0.1	≈ 32	200
				11.8 ± 3.0	≈ 2×10^{-4}	0.8 ± 0.2	≈ 64	400
				7.4 ± 1.5	≈ 1×10^{-4}	0.5 ± 0.1	≈ 164	600
				8.9 ± 1.5	≈ 2×10^{-4}	0.6 ± 0.1	≈ 171	800
				**[C_4C_1im][NTf_2] (200, 400, 600, 800 ML) / ITO-glass**				
**[C_4C_1im][NTf_2]**	483	≈ 0.09	≈ 33	7.2 ± 1.4	≈ 2×10^{-4}	0.5 ± 0.1	≈ 52	200
				7.2 ± 1.4	≈ 2×10^{-4}	0.5 ± 0.2	≈ 105	400
				5.8 ± 1.4	≈ 2×10^{-4}	0.4 ± 0.1	≈ 196	600
				8.6 ± 1.4	≈ 3×10^{-4}	0.6 ± 0.1	≈ 174	800
				**[C_5C_5im][NTf_2] (200, 400, 600, 800 ML) / ITO-glass**				
**[C_5C_5im][NTf_2]**	488	≈ 0.19	≈ 75	7.7 ± 1.3	≈ 1×10^{-4}	0.6 ± 0.1	≈ 48	200
				10.3 ± 1.3	≈ 1×10^{-4}	0.8 ± 0.1	≈ 71	400
				14.2 ± 3.9	≈ 2×10^{-4}	1.1 ± 0.3	≈ 78	600
				10.3 ± 1.3	≈ 1×10^{-4}	0.8 ± 0.1	≈ 143	800
